# Supplementary material for: Real-space observation of incommensurate spin density wave and coexisting charge density wave on Cr (001) surface
Source: Nat Commun. 2022 Jan 21;13:445. doi: 10.1038/s41467-022-28104-2 (PMC8782872; doi:10.1038/s41467-022-28104-2)
Supplement: Supplementary file 1 — Supplementary Information [file 41467_2022_28104_MOESM1_ESM.pdf]

Supplementary materials of

"Real-space Observation of Incommensurate Spin Density Wave and Coexisting Charge Density Wave on Cr(001) surface"

This supplementary material contains additional data of surface characterization, tunneling spectrum and d*I*/d*V* mapping of Cr (001), included in Figs. S1-S9. Detailed descriptions are in the figure captions.

Contains:

Supplementary Figure S1. **The surface dislocation lines and corresponding d*I*/d*V* map ---------------2**

Supplementary Figure S2. **Spatial dependence of the tunneling spectrum --------------------------------3**

Supplementary Figure S3. **A set of dI/dV maps taken at various energies----------------------------------4**

Supplementary Figure S4. **FFT images and the non-dispersive behavior of SDW/CDW ----------------5**

Supplementary Figure S5. **A data set measured by Pt/Ir tip at *T* = 5.0 K ----------------------------------6**

Supplementary Figure S6. **A data set measured by Pt/Ir tip at *T* = 78 K ------------------------------------7**

Supplementary Figure S7. **A data set measured by Pt/Ir tip at T = 301 K ---------------------------------8**

Supplementary Figure S8. **A sketch of band folding effect ---8**

Supplementary Figure S9. **Additional data of QPI measurement ---9**

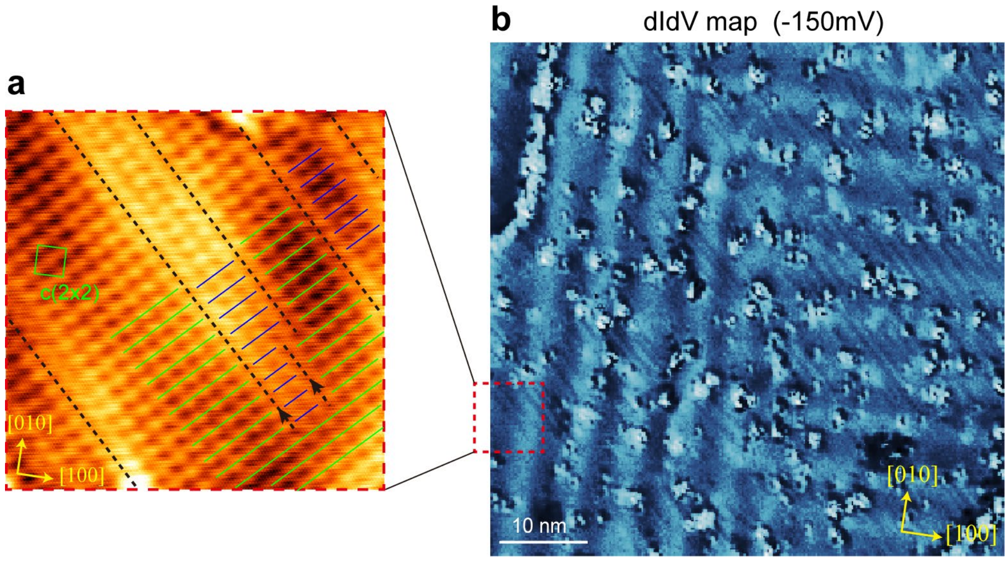

Supplementary Figure S1. The surface dislocation lines and corresponding d*I*/d*V* map (Cr coated tip, *T*= 5.0 K). (**a**) Topographic image (8×8 nm^2) taken at the dashed box area in panel **b**. The atomic c(2×2) lattice and dislocation lines can be seen. The lattice on one side of the dislocation line gradually shifts (starting from the two arrows) with respected to the lattice on the other side, as indicated by the green/blue solid lines. (**b**) d*I*/d*V* map taken by a Cr coated tip (V_b = -150 mV, I = 80 pA, ΔV = 20 mV, 60×60 nm^2). The short stripes along the $1\bar{1}0$ directions are caused by the dislocation lines as shown in panel **a**. They are randomly distributed with no fixed period, and they do not show influence to the long-period SDW modulation.

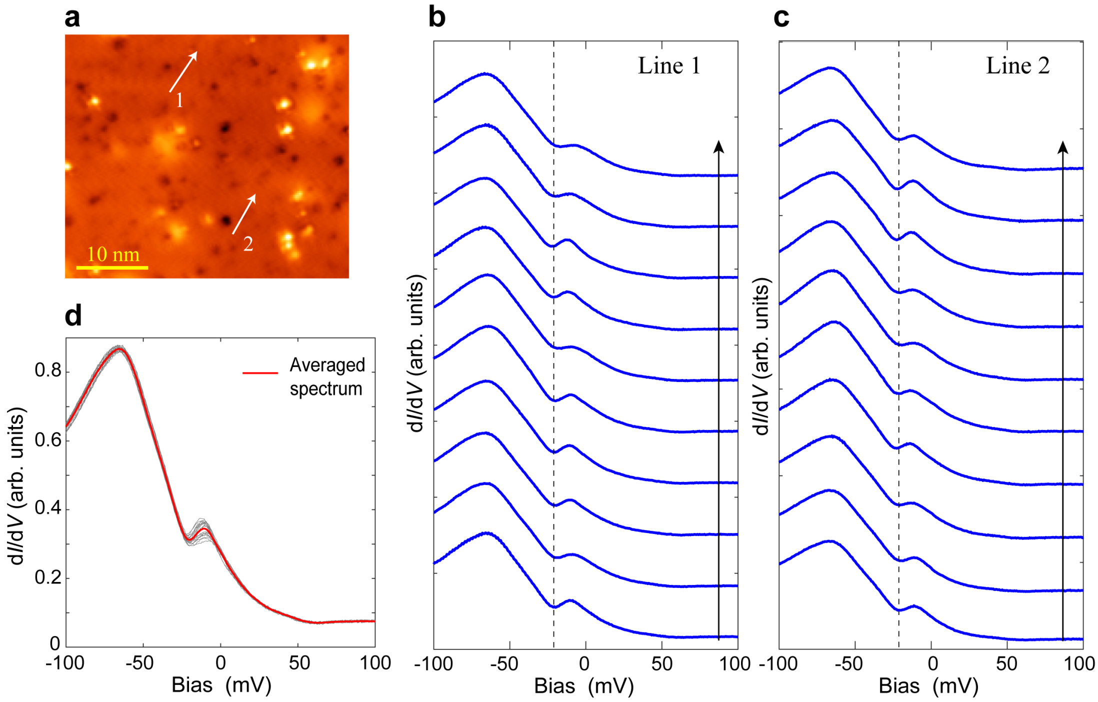

Supplementary Figure S2. Spatial dependence of the tunneling spectrum (Pt/Ir tip, T = 5.0 K). (**a**) Topographic image of Cr(001) (V_b = -30 mV, I = 10 pA). (**b - c**) Series of dI/dV spectra taken along the two arrows shown in panel **a**, respectively (setpoint: V_b = -100 mV, I = 150 pA, ΔV = 5 mV). The DOS dip at E = -22(±1) meV appears in every spectrum. The spectra are shifted vertically. (**d**) A non-shifted plot of all the spectra that shown in panel **b** and **c**, the red curve is averaged spectrum.

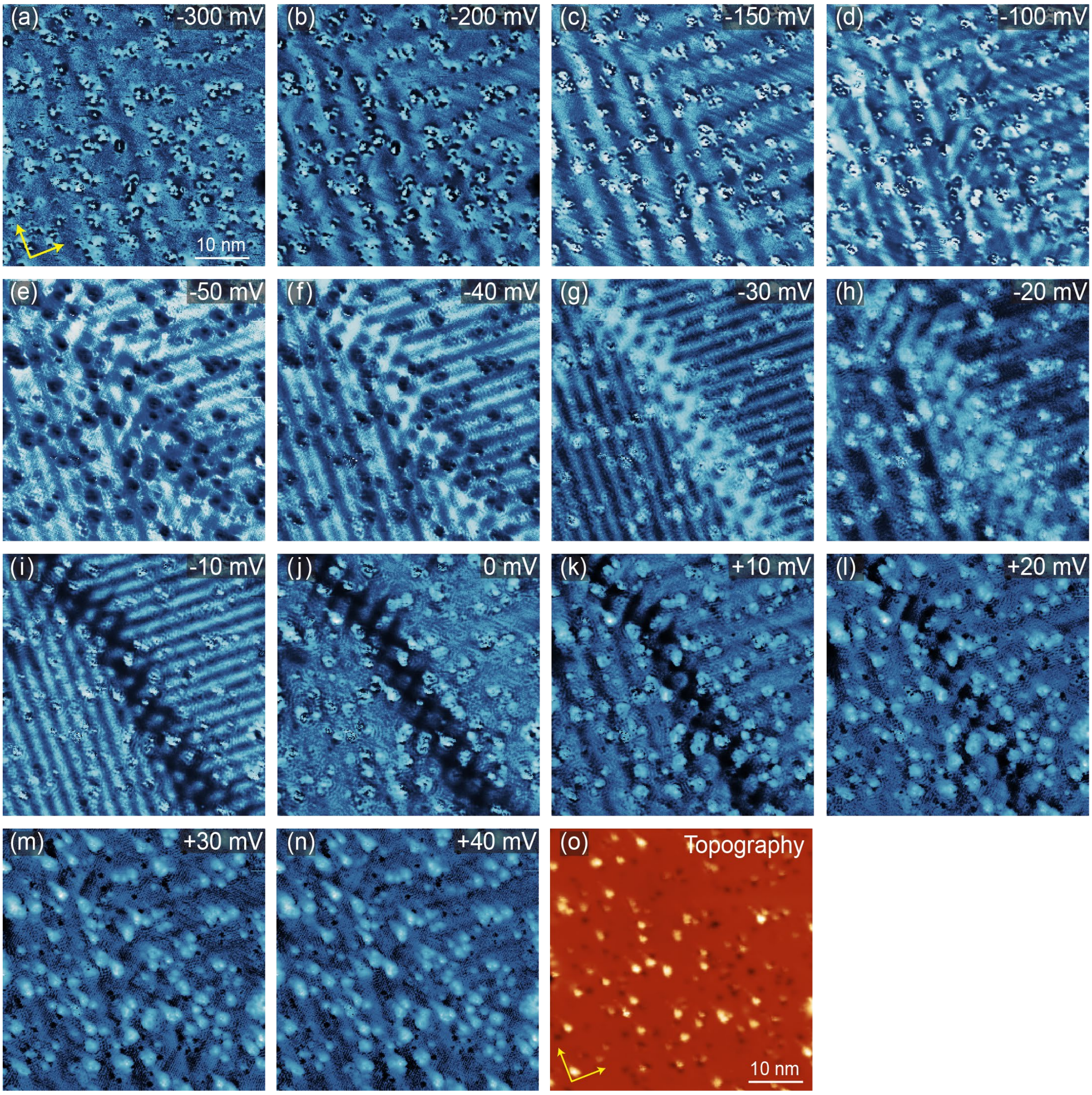

Supplementary Figure S3. (**a-n**) A set of d*I*/d*V* maps taken at various energies with a Cr-coated tip (T = 5.0 K). The mapping energy is labeled in each panel. (**o**) The topographic image of the mapping area. Two SDW/CDW domains can be seen in d*I*/d*V* maps, while the domain structure is unrelated to topography. (Setpoint: I = 150 pA for all the maps. V_b = -300 mV for panel **a**. V_b = -200 mV for panel **b**, V_b = -150 mV for panel **c**, V_b = -100 mV for panel **d**, V_b = -50 mV for panels **e - n**. ΔV = 20 mV for panels **a - b**, ΔV = 15 mV for panel **c**, ΔV = 10 mV for panel **d** and ΔV = 5 mV for panels **e - n**.)

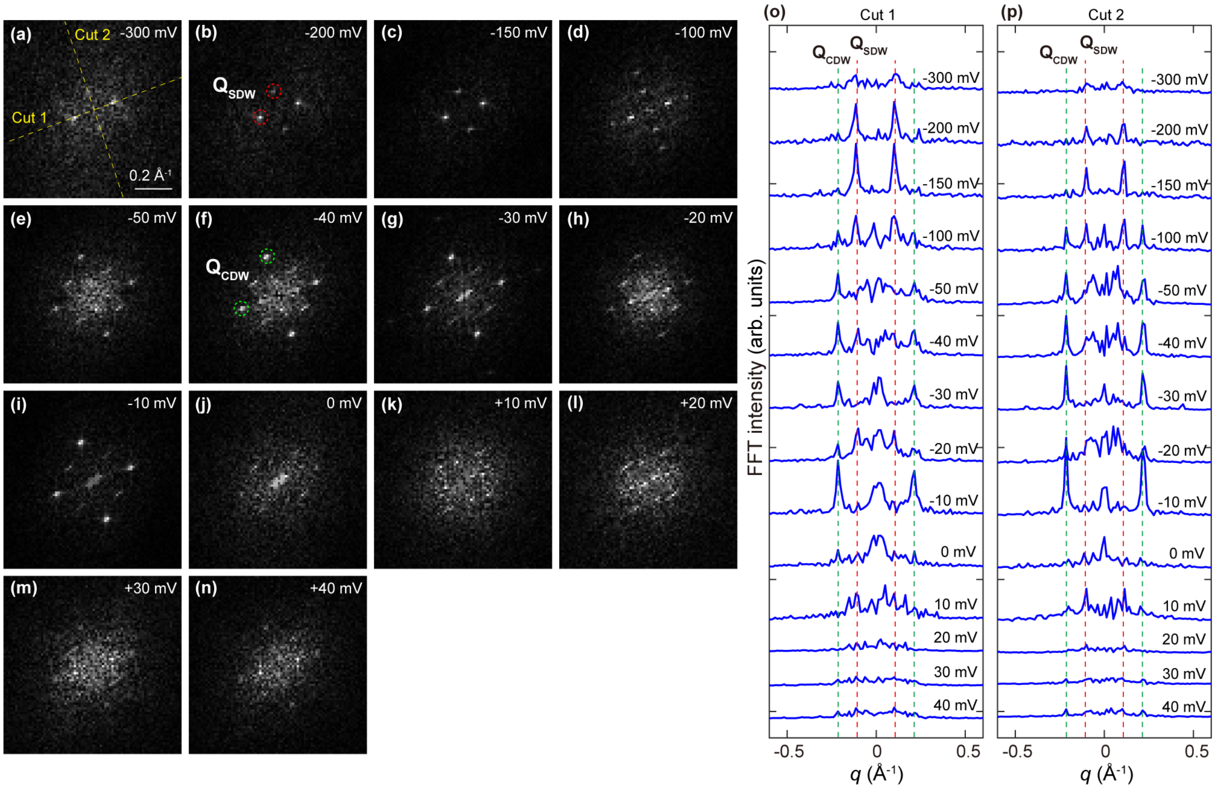

Supplementary Figure S4. FFT images and the non-dispersive behavior of SDW/CDW. (**a - n**) Raw FFT images of the d*I*/d*V* maps shown in Fig. S3(a - n), respectively. Two domains of the SDW/CDW that perpendicular to each other give rise to two sets of SDW/CDW spots in FFT. The red and green dashed circles in panels **b** and **f** indicate the SDW and CDW spots, respectively. (**o - p**) FFT line-profiles at different energies, taken along the two yellow dashed lines marked in panel **a**. The green and red dashed lines indicate the positions of $\mathbf{Q}_{\mathrm{CDW}}$ = 0.21 Å^{-1} and $\mathbf{Q}_{\mathrm{SDW}}$ = 0.105 Å^{-1}, respectively.

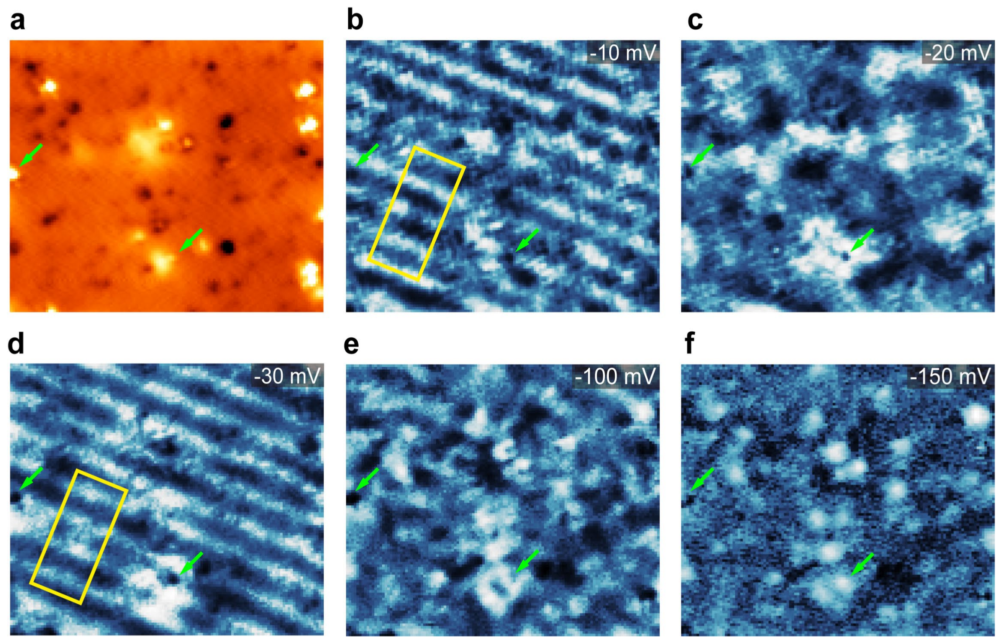

Supplementary Figure S5. A data set measured by Pt/Ir tip at *T* = 5.0 K. (**a**) Topographic image of Cr(001) (29×25 nm^2). (**b - f**) d*I*/d*V* maps taken in the same area as panel **a**. The mapping energies are labelled in each panel. The 3.0 nm period CDW modulations are still present at energies close to E_F, but the SDW modulations are absent in all the maps. The CDW phase inversion between -30 meV and -10 meV can also be seen (indicated by the rectangles at the same position in panels **b** and **d**). (Setpoints: **b**, I = 100 pA, V_b = -10mV, ΔV = 2 mV; **c**, I = 100 pA, V_b = -20mV, ΔV = 3 mV; **d**, I = 100 pA, V_b = -30mV, ΔV = 2 mV; **e**, I = 150 pA, V_b = -100mV, ΔV = 5 mV; **f**, I = 150 pA, V_b = -150mV, ΔV = 5 mV). The two green arrows in each panel point to two defects and their induced LDOS features in d*I*/d*V* maps.

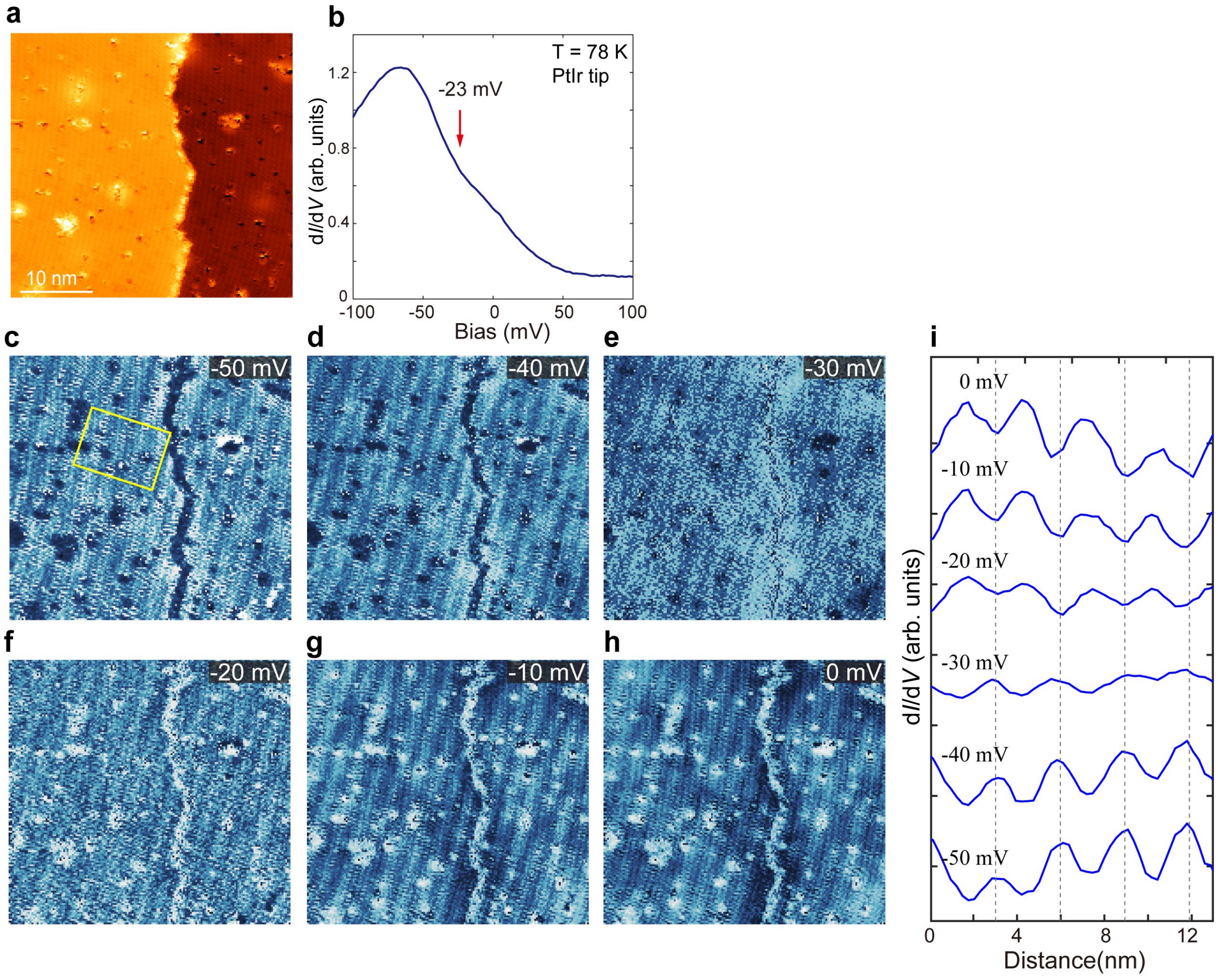

Supplementary Figure S6. A data set measured by Pt/Ir tip at *T* = 78 K. (**a**) Topographic image of the Cr(001) surface (V_b = -40 mV, I = 50 pA). (**b**) Spatially averaged d*I*/d*V* spectrum (setpoint: I = 100 pA, V_b = -100 mV, ΔV = 1 mV). A kink feature is observed around -23 meV. (**c - h**) d*I*/d*V* maps taken in the same area of panel **a** and at various energies (setpoint: I = 50 pA, V_b = -50 mV, ΔV = 8 mV). (**i**) Line profiles of the d*I*/d*V* maps (averaged within the region marked in panel **c**). A phase inversion can be seen between V_b = -30 mV and -20 mV.

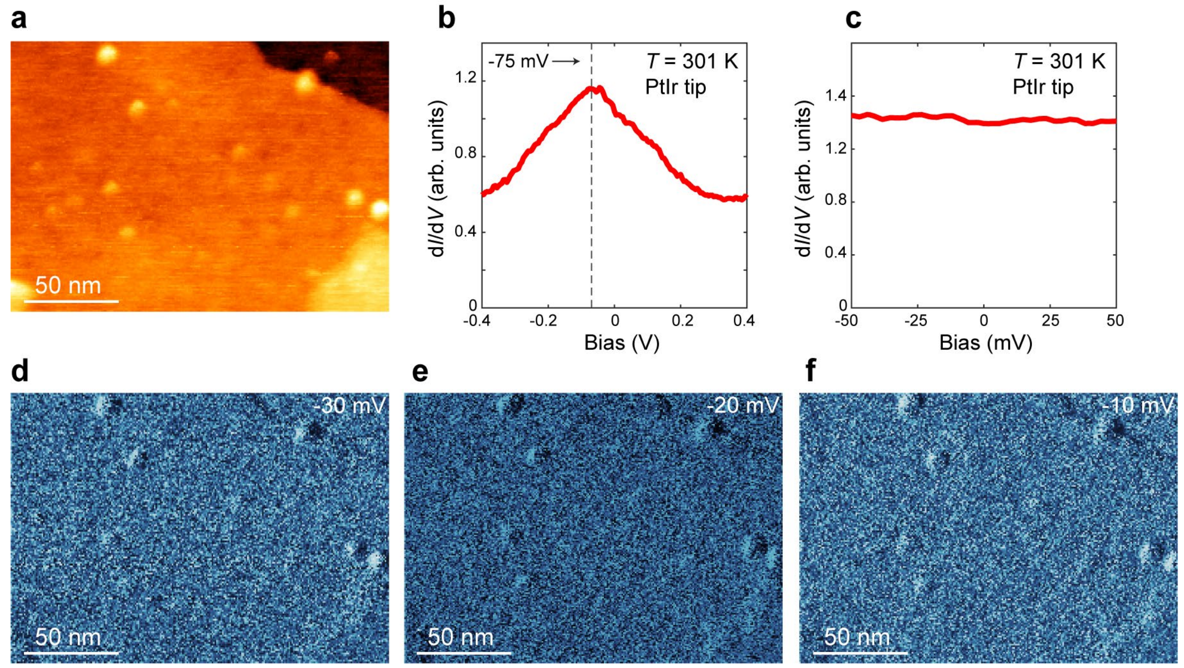

Supplementary Figure S7. A data set measured by Pt/Ir tip at T = 301 K. (**a**) Topographic image of Cr(001) surface (V_b = -30 mV, I = 100 pA). (**b - c**) Averaged dI/dV spectra measured at defect free area. (setpoint: **b**, I = 100 pA, V_b = -1.0V, ΔV = 10 mV; **c**, I = 100 pA, V_b = -0.2V, ΔV = 1 mV). (**d - f**) Several dI/dV maps taken in the same area as panel **a** (setpoint: I = 100 pA; V_b is labeled in each panel; ΔV = 3 mV). No CDW modulation is observed.

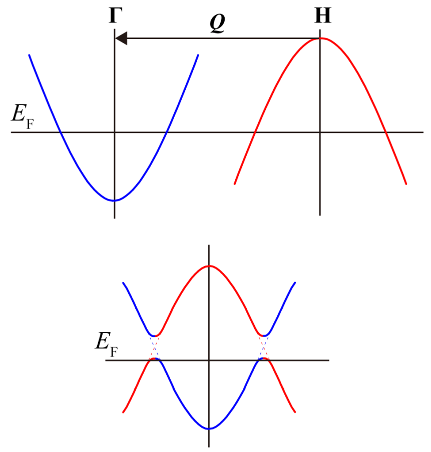

Supplementary Figure S8. A sketch showing the band folding from H to Γ which can induce a hybridization gap above E_F, as the hole pocket at H is slightly larger than the electron pocket at Γ (based on the Fermi surface of Cr reported in L. Mattheiss, Phys. Rev. 139, 1893A (1965) and D. Laurent et al., PRB 23, 4977(1981)). The folding vector between Γ and H points is **Q** = 2π/a.

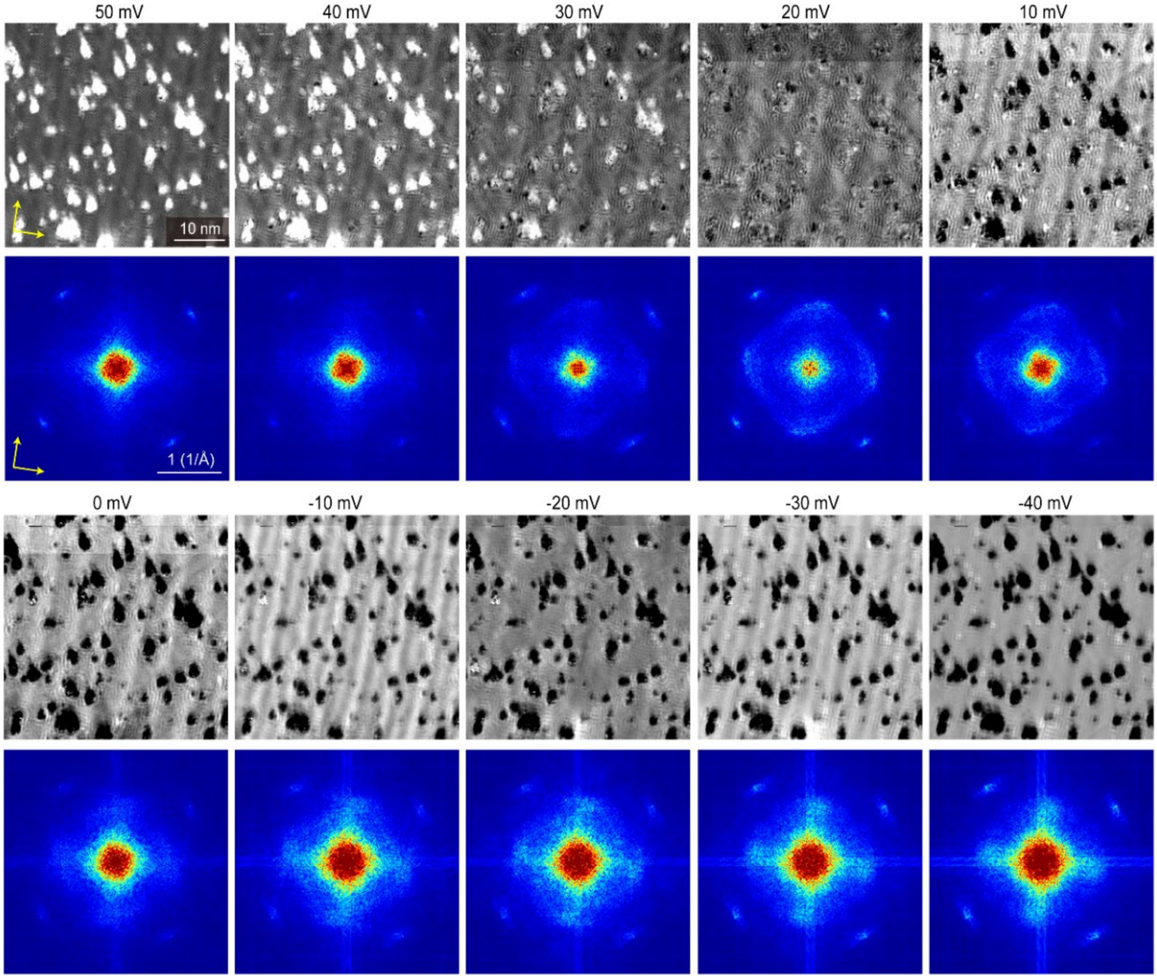

Supplementary Figure S9. Additional data of QPI measurement: A set of d*I*/d*V* maps and corresponding FFT images taken by a Cr coated tip at T = 5.0 K, which show QPI patterns together with the SDW/CDW modulations. The mapping energy is labeled in each panel and the FFTs are four-fold symmetrized. (Setpoint: I = 100 pA, V_b = 50mV, ΔV = 5 mV)
